# Supplementary material for: System-Specific Parameter Optimization for Nonpolarizable and Polarizable Force Fields
Source: J Chem Theory Comput. 2024 Jan 27;20(3):1448–64. doi: 10.1021/acs.jctc.3c01141 (PMC10867808; doi:10.1021/acs.jctc.3c01141)
Supplement: Supplementary file 1 — ct3c01141_si_001.pdf [file ct3c01141_si_001.pdf]

# – *Supporting Information* –

System-Specific Parameter Optimization for Nonpolarizable and Polarizable Force Fields

Xiaojuan Hu,[*,†] Kazi S. Amin,[*,‡] Markus Schneider,[†] Carmay Lim,[¶,§] Dennis Salahub,[*,‖] and Carsten Baldauf[*,†]

†*Fritz-Haber-Institut der Max-Planck-Gesellschaft, Faradayweg 4-6, 14195 Berlin, Germany*

‡*Centre for Molecular Simulation and Department of Biological Sciences, University of Calgary, 2500 University Drive NW, Calgary, Alberta T2N 1N4, Canada*

¶*Institute of Biomedical Sciences, Academia Sinica, Taipei 115, Taiwan*

§*Department of Chemistry, National Tsing Hua University, Hsinchu 300, Taiwan*

‖*Centre for Molecular Simulation and Department of Chemistry, University of Calgary, 2500 University Drive NW, Calgary, Alberta T2N 1N4, Canada*

E-mail: xhu@fhi-berlin.mpg.de; kazi.amin@ucalgary.ca; dsalahub@ucalgary.ca; baldauf@fhi-berlin.mpg.de

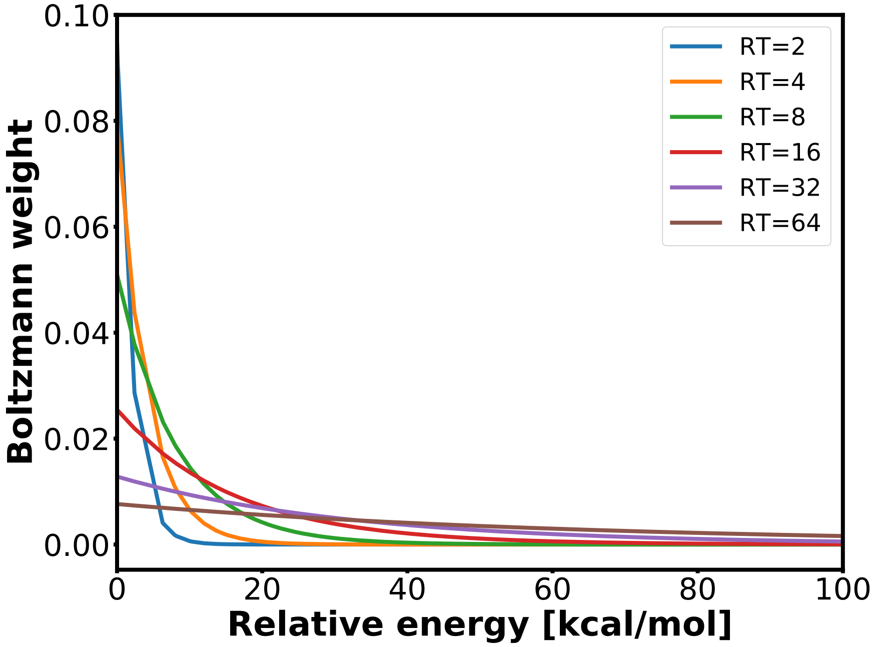

Figure S1: Boltzmann-type weights vs. relative QM energies at various RTs of $AcCys^{-}NMe+Zn^{2+}$ system. All RT values are in units of kcal/mol.

Table S1: Atom types in HisD+Zn^{2+} and Cys^{-}+Zn^{2+}.

HisD+Zn^{2+}		Cys^{-}+Zn^{2+}	
Atom	Atom type	Atom	Atom type
C	2177	C	1177
CA	2166	CA	1166
CB	2446	CB	1148
CD2	2448	H	1183
CE1	2447	HA	1086
CG	2449	HB2	1085
H	2183	HB3	1085
HA	2086	N	1180
HB2	2085	O	1178
HB3	2085	SG	1142
HD1	2445		
HD2	2091		
HE1	2092		
N	2180		
ND1	2444		
NE2	2452		
O	2178		
Zn	834		

Table S2: The optimized LJ parameters. The corresponding atoms for the atom types are shown in Table S1. Epsilon = 0 means the LJ interaction is neglected. LASSO tends to focus on only important factors while neglecting insignificant ones.

Type1	Type2	Sigma (nm)	Epsilon (kJ/mol)
2178	834	0.31933	0.00024413
2448	834	0.331094	0
2183	834	0.32642	0.001277
2446	834	0.32934	0.03138
2177	834	0.330867	0
2092	834	0.288564	0
2091	834	0.288564	0
2180	834	0.319954	0
2447	834	0.329767	0
2444	834	0.31992	0.25885
2445	834	0.29663	6.7250
2085	834	0.294726	0
2086	834	0.294726	0
2184	834	0.325209	0
2452	834	0.32598	0.00153
2166	834	0.331252	0.01205
2449	834	0.33125	0.01205

Table S3: The CTPOL parameters. The a and b are parameters in eq. 12, r is the cutoff distance. The correction factor k in eq. 12 is set as 3.418.

Type	Polarizability (nm^3)	a	b	r (nm)
1142	0.002668	-1.037	0.323	0.312
1178	0.000729	-0.246	0.072	0.294
1180	0.00093	-0.478	0.129	0.270
2178	0.000721	-2.667	0.722	0.271
2180	0.000901	-0.635	0.172	0.270
2452	0.000952	-0.593	0.193	0.325
2444	0.000879	-2.424	0.843	0.348
444	0.000879			
452	0.000952			
834	0.004383			
166/2166/1166	0.001454			
447/2447	0.001341			
448/2448	0.001416			
80	0.001316			
1177	0.001473			
446/2446	0.001397			
177/2177	0.001441			
178	0.000721			
184	0.001292			
449/2449	0.001446			
180	0.000901			
1148	0.001475			
96	0.000724			
250	0.001394			
246	0.000906			
235	0.001339			
81/82	0.001431			
243	0.000864			
94	0.001457			
108	0.001497			
214	0.000858			
213	0.001685			
230	0.000810			
179	0.000904			
165	0.001425			
90	0.001471			
251	0.001417			
216	0.001504			
109	0.000711			
99	0.001439			
245	0.001410			

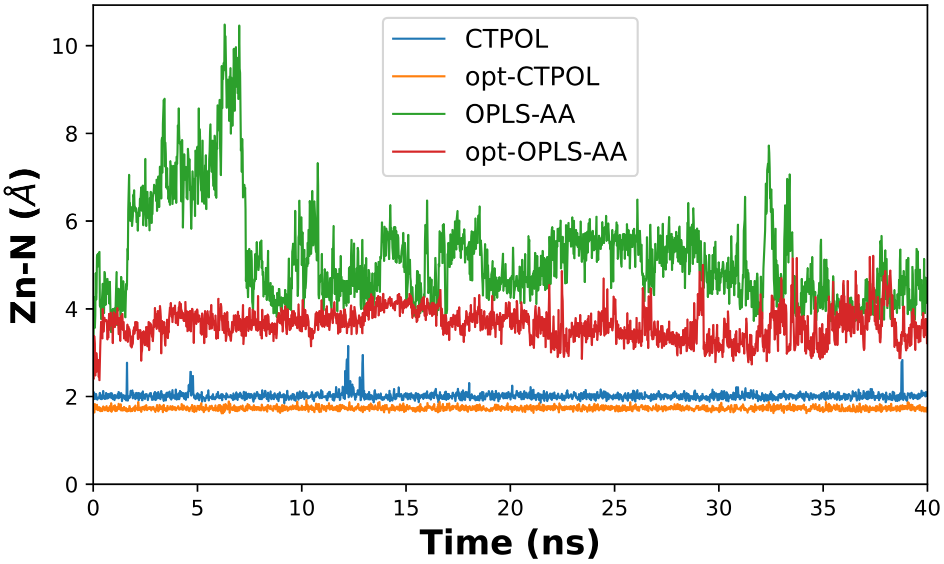

Figure S2: Average of the two Zn-N distances, where the N are the NE2 atoms of the two histidines in the binding site, as a function of time.

Table S4: PDB IDs of Zn fingers. N4HC denotes number of Zn binding sites with 4 His and Cys residues, whereas N2H2C denotes the number of Zn binding sites with exactly 2 His and 2 Cys. The last column denotes the distance of the closest water molecule to the Zn ion.

PDBid	Zn_sites	N4HC	N2H2C	Min H2O dist
1MEY	8	7	7	4.38
4QF3	4	4	0	3.98
6UEI	4	4	0	4.24
6UEJ	4	4	0	4.30
2PUY	4	4	0	4.35
6FI1	4	4	0	9.00
6FHQ	4	4	0	4.04
5YC3	2	2	0	6.49
3T7L	2	2	0	4.41
3U9G	4	4	0	4.26
4Q6F	8	8	0	4.32
3IUF	1	1	1	5.42
4BBQ	8	8	0	4.44
5YC4	2	2	0	6.60
5Y20	2	2	0	5.66

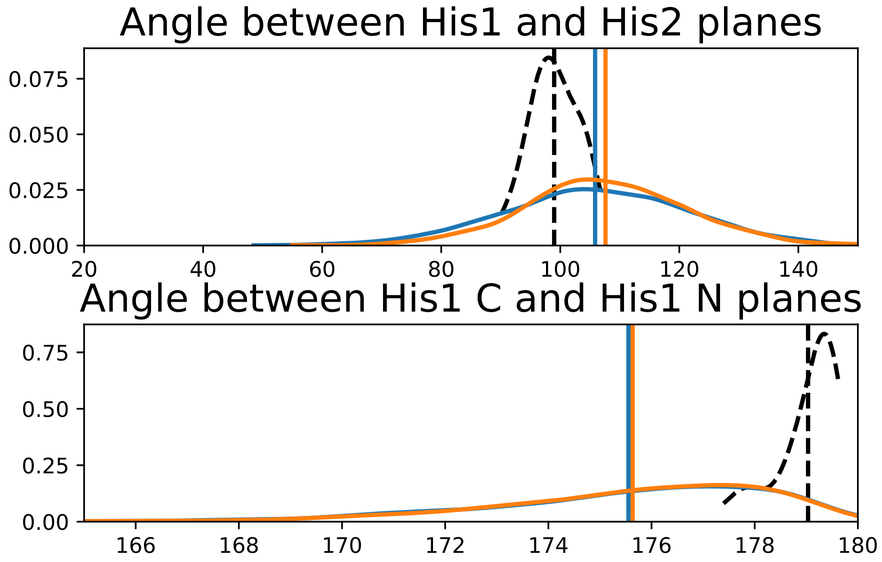

Figure S3: Probability distributions of select dihedral angles. (top) The dihedral angle between the two coordinating histidine planes. The planes were determined using the CG, CD, and CE atoms of histidine. (bottom) The dihedral angle between plane defined by His1 CG, CD, and CE1 atoms, and plane defined by His1 CG, ND, and NE atoms. This is to check for internal distortion of the plane. The values are close to 180 (instead of 0) because one set of atoms goes clockwise, and the other counter clockwise, when defining the planes.
